# Derivatization with 2-hydrazino-1-methylpyridine enhances sensitivity of analysis of 5α-dihydrotestosterone in human plasma by liquid chromatography tandem mass spectrometry

**DOI:** 10.1016/j.chroma.2021.461933

**Published:** 2021-03-15

**Authors:** Abdullah MM Faqehi, Scott G Denham, Gregorio Naredo, Diego F Cobice, Shazia Khan, Joanna P Simpson, Ghazali Sabil, Rita Upreti, Fraser Gibb, Natalie ZM Homer, Ruth Andrew

**Affiliations:** a University/British Heart Foundation Centre for Cardiovascular Science, United Kingdom; b Mass Spectrometry Core, Edinburgh Clinical Research Facility, Queen's Medical Research Institute, University of Edinburgh, 47 Little France Crescent, Edinburgh EH16 4TJ, United Kingdom

**Keywords:** Testosterone, Androstenedione, 5α-Dihydrotestosterone, Liquid chromatography mass spectrometry, Derivatization

## Abstract

Liquid Chromatography tandem mass spectrometry (LC-MS/MS) is the gold-standard approach for androgen analysis in biological fluids, superseding immunoassays in selectivity, particularly at low concentrations. While LC-MS/MS is established for analysis of testosterone and androstenedione, 5α-dihydrotestosterone (DHT) presents greater analytical challenges. DHT circulates at low nanomolar concentrations in men and lower in women, ionizing inefficiently and suffering from isobaric interference from other androgens. Even using current LC-MS/MS technology, large plasma volumes (>0.5 mL) are required for detection, undesirable clinically and unsuitable for animals. This study investigated derivatization approaches using hydrazine-based reagents to enhance ionization efficiency and sensitivity of analysis of DHT by LC-MS/MS. Derivatization of DHT using 2-hydrazino-1-methylpyridine (HMP) and 2-hydrazino-4-(trifluoromethyl)-pyrimidine (HTP) were compared. A method was validated using an UHPLC interfaced by electrospray with a triple quadruple mass spectrometer , analyzing human plasma (male and post-menopausal women) following solid-phase extraction. HMP derivatives were selected for validation affording greater sensitivity than those formed with HTP. HMP derivatives were detected by selected reaction monitoring (DHT-HMP *m/z* 396→108; testosterone-HMP *m/*z 394→108; androstenedione-HMP *m/z* 392→108). Chromatographic separation of androgen derivatives was optimized, carefully separating isobaric interferents and acceptable outputs for precision and trueness achieved following injection of 0.4 pg on column (approximately 34 pmol/L). HMP derivatives of all androgens tested could be detected in low plasma volumes: male (100 µL) and post-menopausal female (200 µL), and derivatives were stable over 30 days at -20°C. In conclusion, HMP derivatization, in conjunction with LC-MS/MS, is suitable for quantitative analysis of DHT, testosterone and androstenedione in low plasma volumes, offering advantages in sensitivity over current methodologies.

## Highlights

• Quantitative analysis of low abundance androgens in human plasma.
• Quantitation of androgens over physiological range in men and post-menopausal women.
• Use of hydrazine derivatives improves analytical sensitivity.

## 1 Introduction

Immunoassays have been widely used to quantify androgens, e.g. across the range of circulating concentrations of testosterone (0.1-37.1 nmol/L) and androstenedione (0.2-8.8 nmol/L) [1], but analytical selectivity at low concentrations (< 1.7 nmol/L) is poor due to interference from endogenous isomers and other steroids in the biological matrix [2]. This presents a particular problem when measuring low abundance bioactive 5α-reduced androgens, 5α-dihydrotestosterone (DHT) and 5α-dihydroandrostanedione (DHA), with plasma concentrations of DHT of only 0.1-1.9 nmol/L in men and 0.1-1.0 nmol/L in women over 16 years [3]. Thus immunoassays cannot be used reliably in males with anti-androgen therapy, and in children and women [4], [5], [6]. Moreover, many diseases and lifestyle factors are associated with low androgens. For example, testosterone levels decrease with obesity [7], smoking [8] and diseases such as type 2 diabetes mellitus [9,10]. Androstenedione levels are even lower in post-menopausal women receiving estrogen therapy [11].

Recently the Endocrine Society published recommendations regarding measuring testosterone in clinical samples [4]. Accordingly, liquid chromatography-tandem mass spectrometry (LC-MS/MS) is already replacing immunoassays in the analysis of testosterone in clinical practice [12,13] and is also of value in preclinical and veterinary studies [14], [15], [16]. However, even with tandem MS coupled to ultra-high-performance LC (UHPLC) [13,17] and other technical innovations such as UHPLC-Q-Orbitrap [14,18] and LC-Microchip technology [17], values for DHT concentrations are often below the limit of detection of analysis in small volumes of human plasma and serum. Thus, large plasma volumes (>500 µL) are required for detection, undesirable clinically and unsuitable for pediatric patients and small animals.

Derivatization of the ketone or hydroxyl group within the androgen [19], [20], [21], [22], [23], [24], [25], [26] may improve sensitivity by introducing a chargeable or permanently charged group, changing efficiency of ionization, fragmentation and retention. LC-MS/MS methods using derivatization often demonstrate improved limits of quantitation (LOQs) by several orders of magnitude [25].

Previous derivatization reagents employed for androgen analysis include picolinic acid [27,28], fusaric acid [29], isonicotinoyl azide [25], hydroxylamine [30], [31], [32], [33], methoxylamine [34], 2-fluoro-1-methylpyridinium-*p*-toluenesulfonate (FMP-TS) [35,36]. In addition, hydrazine-based reagents: 1-(carboxymethyl) trimethylammonium chloride hydrazide (Girard T) [37] and 2-hydrazinopyridine (HP) have been used to increase sensitivity [25,38] and further reagents have been explored but not been fully validated or applied to biological samples. 2-Hydrazino-1-methylpyridine (HMP) has a permanently charged moiety (quaternary ammonium) and its reaction with the oxo-steroids (e.g. testosterone, DHT and DHEA) was used in LC-MS/MS analysis of human prostatic tissue, saliva, rat serum and brain [20,21,[39], [40], [41]]. However, HMP derivatives form several isomers [21], in some cases 4, and previous chromatographic methods have struggled to resolve all isomers from potential isobaric interferences. Specifically, testosterone enriched naturally with two heavy isotopes (e.g. deuterium and/or ^13^C) interferes with DHT analysis, at nominal mass. Weng et al [14] proposed the use of 2-hydrazino-4-(trifluoromethyl)-pyrimidine (HTP) derivatives as an alternative since the isomers co-elute causing less chromatographic complexity.

We aimed to overcome the chromatographic problems faced with the hydrazine derivatives and validate an analytical approach to detect low abundance DHT by derivatization in plasma from men and post-menopausal women.

## 2 Materials and methods

### 2.1 Instrumentation

A QTrap 5500 triple quadrupole MS (Sciex, Warrington, UK) coupled to an Acquity™ Classic Ultra Performance LC (Waters Corporation, Milford, USA), was operated using Analyst software version 1.5.1. Confirmation of accurate mass of HMP was performed by direct infusion into a 12 T SolariX dual source Fourier Transform Ion Cyclotron Resonance MS (FTICR MS; Bruker Daltonics, MA, US), operated with SolariX control v1.5.0 (build 42.8) software. The accurate masses of HMP derivatives were assigned by infusing into a Synapt G2-Si Q-ToF MS (Waters Corporation, Milford, USA) with an electrospray (ESI) ionization source.

### 2.2 Standards and solvents

#### 2.2.1 Commercial sources

Testosterone, androstenedione, DHT, DHA, epitestosterone (EpiT), dehydroepiandrosterone (DHEA), formic acid (FA) ≥98%, hydrazine monohydrate, dichloromethane (DCM), trifluoroacetic acid (TFA), ammonium acetate and 2-fluoro-1-methylpyridinium-*p*-toluenesulfonate (FMP-TS) were from Sigma-Aldrich, (Dorset, UK). 2,3,4-^13^C_3-_Testosterone (^13^C_3_-T), 99%, 2,3,4-^13^C_3_-androstenedione (^13^C_3_-A4), ≥98%, 2,3,4-^13^C_3_-5α-dihydrotestosterone (^13^C_3_-DHT), ≥97% were from IsoSciences (Philadelphia, USA). Certified solutions of testosterone, androstendione (both 1 mg/mL in acetonitrile) and DHT (1 mg/mL in methanol) and ^13^C_3_-T and ^13^C_3_-A4 (100 µg/mL in acetonitrile) were from Cerilliant (Texas, USA). HPLC grade glass distilled solvents (methanol, acetonitrile, acetone, *n*-hexane, DCM and water) and LC-MS grade solvents (methanol, acetonitrile and water) were from Fisher Scientific UK Limited (Leicestershire, UK).

#### 2.2.2 Synthesis of 2-hydrazino-1-methylpyridine (HMP)

**Fig. 1:**
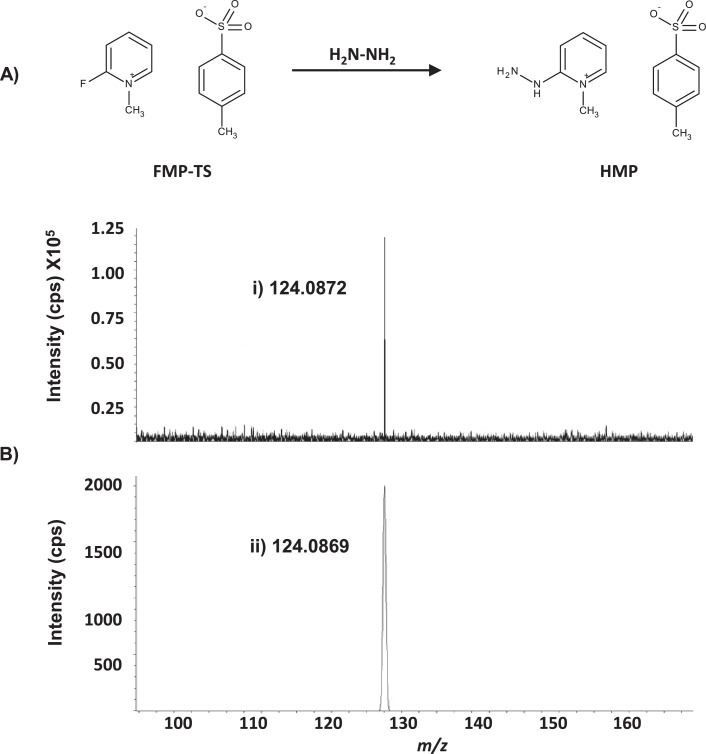
(A) Synthetic reaction of HMP from FMP-TS; 2-fluoro-1-methylpyridinium *p*-toluene sulfonate (FMP-TS) and 2-hydrazino-4-(trifluoromethyl)-pyrimidine (HMP) (B) Exact mass of HMP was confirmed by FTICR-MS analysis, HMP elemental formula of C_6_H_10_N_3_ and the theoretical mass (i) *m*/*z* 124.0872 were aligned with accurate mass (ii) *m*/*z* 124.0869. Counts per second (cps).

HMP was synthesized from FMP-TS (Fig. 1A), using a method adapted from [21]. Methanol (100 mL) was placed in a flask (~0°C) and hydrazine monohydrate (65% w/v in methanol, 660 µL) added, producing a colourless solution. FMP-TS (1.5 g) was dissolved in methanol (30 mL) and added dropwise, resulting in a yellow solution, which was stirred (0°C, 15 min, and then at room temperature (18-22°C, 60 min)). The solution was concentrated to dryness under vacuum, generating an orange residue. The residue was re-dissolved in hot dichloromethane (DCM (100 mL)) and heated (60°C) with dropwise addition of *n*-hexane (10 mL) to produce a cloudy solution. This was clarified by heating the flask and then stored overnight (4°C, 48 hr). The product was filtered and crystallized twice as described [21] with a 65% (3.9 g) yield of needle-like, colourless crystals, which were stored at -20°C.

Ions of accurate mass of HMP were obtained in full scan (*m/z* 50 -1200) by FTICR-MS in ESI positive mode, with capillary voltage (4500 V), nebulizer pressure (1.2 bar) and source temperature (200°C). The elemental composition of the HMP product was confirmed with *m*/*z* 124.0869, and a Δppm of 2.4 from the theoretical mass (124.0872 Da; C_6_H_10_N_3_) (Fig. 1A and B).

### 2.3 Sources of biological samples

Pooled male and post-menopausal female human plasma used in method development and validation were from TCS Biosciences (Buckingham, UK), obtained from healthy donors in approved blood collection centers and stored at -20°C. Plasma was prepared from whole blood collected into anticoagulant (citrate phosphate dextrose adenine, CPDA-1). DC Mass Spect Gold® Serum was from Merck Life Sciences (Darmstadt, Germany), which is plasma depleted of steroids and hormones. For method application, male and post-menopausal female human plasma samples were obtained from subjects participating in experimental medicine studies for which local ethical approval had been obtained; 17 post-menopausal women (58-60 years) and 42 men (21-85 years) [42], [43], [44].

### 2.4 Standard solutions

For chromatographic assessment, androgens (testosterone, androstenedione, DHT, DHA, DHEA and EpiT) and internal standards (IS; ^13^C_3_-T, ^13^C_3_-A4 and ^13^C_3_-DHT, 1 mg) were individually dissolved in methanol (1 mL) and stored at -20°C. Stock solutions (1 mg/mL) were diluted by 100 fold serial dilutions in methanol to achieve 1 ng/mL and further by 10 fold to 0.1 ng/mL solutions on the day of use. Data were validated against certified standard solutions, when available (2.2.1), diluted by 100 fold serial dilution in methanol from 100 μg/mL to achieve 10 ng/mL solutions

### 2.5 Generation of HMP and HTP derivatives and mass spectral characterization

#### 2.5.1 Optimization of reaction conditions

Incubation temperatures (25-80°C), time (5-120 min) and reaction volume (100-1000 µL) were evaluated for both derivatives, using a range of concentrations of reagent (HMP 0.1 - 2 and HTP 0.05 - 5 mg/mL) freshly prepared prior to reaction.

##### 2.5.1.1 Optimized method for formation of HMP derivatives

HMP (100 µL; 0.5 mg/mL in methanol, containing FA (1% v/v)) was added to standards (1 ng)/extract and vortexed (10 s). Incubation was performed with HMP (60°C, 15 min) and the reaction quenched by dilution in methanol (50 μL).Solvents were evaporated under oxygen-free N_2_ (OFN; 60°C) and the sample reconstituted in initial mobile phase AND THE SAMPLE (50 µL) [21].

##### 2.5.1.2 Optimized method for formation of HTP derivatives

HTP (100 µL; 0.1 mg/mL in acetonitrile, containing TFA (0.05% v/v)) was added to standards (1 ng)/extract and then vortexed (10 s). Incubation was performed (60°C, 30 min) and then the mixture cooled (ice bath, 5 mins). Solvents were evaporated under OFN (60°C) and HTP derivatives reconstituted in initial mobile phase (50 µL).

#### 2.5.2 MS tuning of derivatives

Precursor and product ions for quantitative analysis were identified by directly infusing individual androgen derivatives (1 µg/mL in methanol, diluted 1:10 in their respective mobile phases (2.6)) into a QTrap 5500 triple quadrupole MS, operated in positive ion ESI mode with entrance potential (10 V). Precursor ions for HMP and HTP derivatives were selected in Q1 (Tables 1 and 2 respectively), and conditions for multiple reactions monitoring (MRM) optimized by autotuning using Analyst software (Sciex, Warrington, UK). Q3 was operated in scanning mode *m/z* 50 below and above the mass of the precursor ion of interest. Positive ESI source conditions were optimized to generate transitions with highest sensitivity of quantifier and qualifier ions (Tables 1 and 2), established in conjunction with curtain gas (20, 25 m Torr), ion gas 1 (40, 45 mTorr) and gas 2 (25, 20 m Torr), collision gas (medium, high), ion spray (4500, 4000 V) and temperature (650, 600°C) for HMP and HTP respectively.

**Table 1:** Optimized tuning conditions for analysis of HMP derivatives of androgens. Voltage (V); testosterone (T), androstenedione (A4), 5α-dihydrotestosterone (DHT), 5α-dihydroandrostanedione (DHA), epitestosterone (EpiT), dehydroepiandrosterone (DHEA), 2,3,4-^13^C_3-_testosterone (^13^C_3_-T), 2,3,4-^13^C_3_-androstenedione (^13^C_3_-A4), 2,3,4-^13^C_3_-5α-dihydrotestosterone (^13^C_3_-DHT); quantifier (*) and qualifier (^$^).

Analyte	Precursor ion *m/z*	Product ions *m/z*	De-clustering potential (V)	Collision energy (V)	Collision cell exit potential (V)
*T*	394	108*	36	49	14
		81^$^		87	10
*A4*	392	108*	51	51	8
		81^$^		87	10
*DHT*	396	108*	46	47	12
		81^$^		85	10
*DHA*	394	108*	66	43	20
		81^$^		91	10
*EpiT*	394	108*	26	57	12
		81^$^		85	8
*DHEA*	394	109*	101	35	12
		81^$^		57	14
*^13^C_3_-T*	397	108*	51	69	10
		81^$^		89	12
*^13^C_3_-A4*	395	108*	66	61	10
		81^$^		83	10
*^13^C_3_-DHT*	399	108*	56	47	14
		81^$^		87	10

**Table 2:** Optimized tuning conditions for analysis of HTP derivatives of androgens. Voltage (V); testosterone (T), androstenedione (A4), 5α-dihydrotestosterone (DHT), 5α-dihydroandrostanedione (DHA), epitestosterone (EpiT), dehydroepiandrosterone (DHEA), 2,3,4-^13^C_3-_testosterone (^13^C_3_-T), 2,3,4-^13^C_3_-androstenedione (^13^C_3_-A4), 2,3,4-^13^C_3_-5α-dihydrotestosterone (^13^C_3_-DHT); quantifier (*) and qualifier (^$^).

Analyte	Precursor ion *m/z*	Product ions *m/z*	De-clustering potential (V)	Collision energy (V)	Collision cell exit potential (V)
*T*	449	257*	26	49	22
		269^$^		49	34
*A4*	447	269*	41	47	12
		257^$^		45	12
*DHT*	451	288*	36	39	12
		164^$^		49	18
*DHA*	449	286*	46	37	24
		164^$^		43	16
*EpiT*	449	257*	36	49	22
		269^$^		49	34
*DHEA*	449	286*	81	37	24
		164^$^		43	16
*^13^C_3_-T*	452	272*	41	47	30
		260^$^		47	4
*^13^C_3_-A4*	450	260*	56	47	30
		272^$^		47	4
*^13^C_3_-DHT*	454	294*	66	39	28
		164^$^		45	20

The identity of HMP derivatives was confirmed by accurate mass measurement using a Synapt G2-Si qTOF MS in resolution mode. Ions were isolated in full scan (*m/z* 50 -1200) in ESI positive mode (source conditions: curtain gas (35 m Torr), collision gas (40 V), ion spray (3000 V) and temperature (100°C)).

### 2.6 Chromatographic conditions for resolution of androgen derivatives

#### 2.6.1 HMP derivatives

Separation of HMP derivatives was achieved over 10 minutes using an Acquity UPLC® BEH C_18_ column (50 × 2.1 mm, 1.7 µm, Waters Corporation, Milford, USA). The aqueous phase was ammonium acetate (5 mM) and the organic phase methanol:acetonitrile (50:50 v/v). Flow (0.5 mL/min) was diverted to waste between 0-2 and 8-10 min. The optimized method began with an initial high aqueous solvent system (90 aqueous:10 organic), sustained for 1 min followed by a rapid gradient over 1 min to achieve (50:50) conditions. Then, a slower gradient was performed for 5 min to (45:55), followed by a rapid gradient to achieve (10:90), over 1 min. The gradient was then returned to starting conditions (90:10) over 1 min and sustained for 2 min to re-equilibrate the column. The column and autosampler temperatures were 50°C and 10°C respectively. Injection volume was 20 µL in partial loop needle overfill mode.

#### 2.6.2 HTP derivatives

Separation of HTP derivatives was performed on an Acquity UPLC® BEH C_18_ column (150 × 2.1 mm, 1.7 µm, Waters Corporation, Milford, USA) with a total run time of 20 minutes at 50°C. Mobile phase comprized aqueous (ammonium acetate (5mM)) and organic (methanol:acetonitrile, 35:65). Flow (0.3 mL/min) was diverted to waste between 0-10 and 18-20 min. A high aqueous solvent system (90 aqueous:10 organic) was sustained for 1 min followed by a rapid gradient over 1 min to achieve (40:60). Conditions were maintained for 10 min, followed by a slow gradient for 5 min to achieve a high organic phase (10:90), executed over 1 min. The gradient was then returned to starting conditions (90:10) over 1 min and sustained for 2 min to re-equilibrate the column.

### 2.7 Optimized extraction method

Aliquots of male (100 µL) and post-menopausal female plasma (200 µL) were subject to centrifugation (8000 g, 4°C, 20 min). Volumes were adjusted with water (to 1 mL), enriched with internal standards (100 pg in 10 µL methanol) and mixed. Solid-phase extraction using Oasis® HLB (1 cc/10 mg, Waters) cartridges was performed under gravity. The cartridges were conditioned with methanol (1 mL) followed by water (1 mL) then the samples, previously enriched with IS, loaded (1 mL). The cartridges were washed with methanol/water (5:95; v/v) and steroids eluted in methanol (1 mL). Extracts were reduced to dryness under OFN (60°C) and the residues were derivatized as above (2.5.1).

### 2.8 Assay validation of HMP derivatives

The method was validated for testosterone, androstenedione and DHT but not DHA, which could not be detected in initial screening of biological samples.

#### 2.8.1 Extraction efficiency

Recoveries of standards and IS from each water and plasma were assessed, following extraction from paired samples, pre- and post-spiked with IS (100 pg; *n=6*) prior to derivatization. Mean peak areas of derivatives following extraction in pre-spiked samples were divided by those in the matched post-spiked samples and expressed as a percentage.

#### 2.8.2 Assessment of ion suppression

Ion suppression of signals of derivatives in the presence of extracts of plasma was evaluated by post-spiking IS (100 pg) into extracted plasma *(n=6).* Mean peak areas of derivatives of steroids following extraction from post-spiked plasma were divided by those of mean peak areas of derivatives of unextracted standards of the same amount and expressed as a percentage.

#### 2.8.3 Selectivity

MRM chromatograms were inspected close to the retention times of HMP derivatives for possible interferences by other endogenous compounds in plasma such as DHEA and EpiT. The ratios of quantifier to qualifier ions of HMP derivatives of endogenous steroids and IS were measured in extracts from plasma and compared with those of standards and accepted if within 20%.

#### 2.8.5 Linearity of response

Extracts of blank samples (IS only) and aqueous aliquots containing androgens (1, 2.5, 5, 10, 25, 50, 100, 200 pg) and internal standards (100 pg) were analyzed. Calibration curves were plotted as the peak area ratio (standard/IS) versus amount of androgen. Calibration lines of best fit were acceptable if the regression coefficient, r, was >0.99. Weightings of 1, 1/x and 1/x^2^ were compared. The bias of the calibrators was assessed against certified standards for testosterone, androstenedione and DHT [45] (average inter-assay in-house standard concentration recorded - certified standard concentration X 100). Parallelism of calibration curves was assessed by comparing calibration standards extracted from both water and from steroid-depleted serum.

#### 2.8.4 Limit of detection (LOD) and quantitation (LOQ)

To compare reagents, androgen derivatives (10, 1 ng and 100, 10, 1, 0.1 pg) were analyzed and the Signal/Noise (SNR) calculated from peak areas of steroids and adjacent background noise, with matched time intervals, integrated manually. The LODs and LOQs of HMP derivatives were then evaluated from 6 replicate calibration lines following extraction from water using equations of (3.3 x standard deviation of response)/standard deviation of slope and (10 x standard deviation of response)/Standard deviation of slope, respectively. Replicate aliquots (1 pg; *n=6*)) of androgens and internal standards were subsequently prepared as above and the LOQ re-assessed, as the concentration affording precision and trueness of 20% or less.

#### 2.8.5 Trueness and precision of HMP derivatives

Injector repeatability was tested by re-injecting standards (1 pg). The intra- and inter-assay precision and trueness were assessed using 6 standard aliquots (1, 50 and 200 pg) extracted from water and prepared on the same and different days respectively, alongside a standard curve *(n=6)*. Aliquots of male (100 µL) and post-menopausal female plasma (200 µL) were extracted 6 times on the same day to assess precision.

Precision was calculated as the Relative Standard Deviation (RSD) (standard deviation/mean X 100), and % trueness was the Relative Mean Error (RME) (measured value - theoretical value X 100).

#### 2.8.6 Stability of HMP derivatives

Stability following storage in the auto-sampler (10°C) was evaluated by reinjection of a calibration curve and plasma sample after 24 h. Short-term storage in the freezer (-20 and -80°C) were assessed by injection after storage for 24 h and 7 and 30 days.

### 2.9 Method application for HMP derivatives

Testosterone, androstenedione and DHT were quantified in replicates of human plasma from post-menopausal females and males using the validated approach. The peak areas of analytes in the samples were divided by those of their respective internal standards and interpolated onto the calibration lines to calculate the amount present in each sample volume.

## 3 Results and discussion

### 3.1 Method development

#### 3.1.1 Derivative selection: comparison of HMP and HTP derivatives

**Fig. 2:**
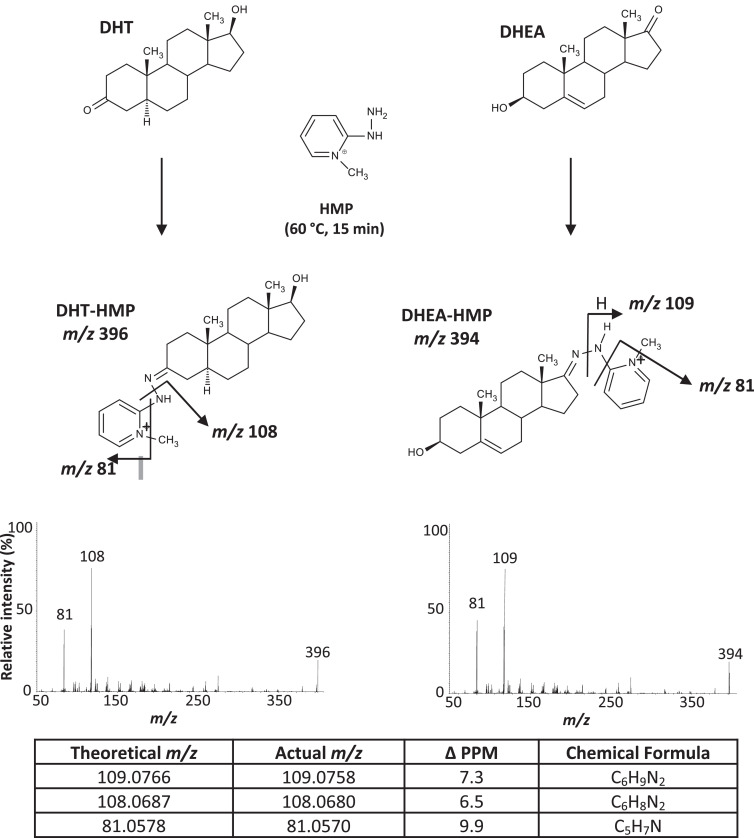
Formation of steroid-HMP derivatives, showing examples of derivatization of DHT (representative of derivatives forming on the A ring) and DHEA (derivatization on the D Ring) and putative fragmentation with product ions scans and confirmation using accurate mass data; difference (Δ), 5α-dihydrotestosterone (DHT), dehydroepiandrosterone (DHEA) and 2-hydrazino-1-methylpyridine (HMP). Products ion scan of further analytes and internal standards can be found in Supplementary Fig. S1. The two product ions selected for analysis were derived from the derivatization reagent.

**Fig. 3:**
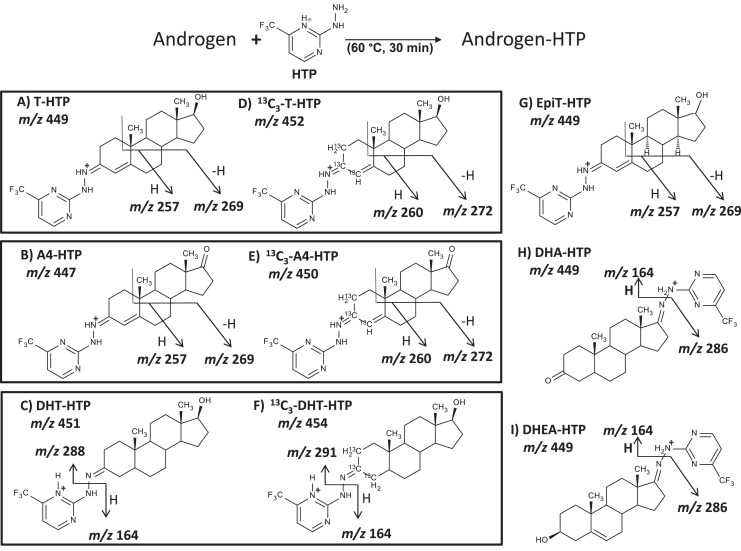
Putative fragmentation of HTP derivatives; testosterone (T), androstenedione (A4), 5α-dihydrotestosterone (DHT), 5α-dihydroandrostanedione (DHA), epitestosterone (EpiT), dehydroepiandrosterone (DHEA), 2,3,4-^13^C_3-_testosterone (^13^C_3_-T), 2,3,4-^13^C_3_-androstenedione (^13^C_3_-A4), 2,3,4-^13^C_3_-5α-dihydrotestosterone (^13^C_3_-DHT) and 2-hydrazino-4-(trifluoromethyl)-pyrimidine (HTP).

Anticipated reactions are shown in Fig. 2, Fig. 3 for HMP and HTP respectively. Reaction efficiency was improved by increasing temperature from 25 to 60°C for both derivativesfor reaction time at 15 min (HMP) and 30 min (HTP). Further improvement was not seen at higher temperatures or with longer incubations. Intensity of response was improved by decreasing the reaction volumes from 500 to 100 µL for both reagents and with optimal concentrations of 0.5 and 0.1 mg/mL for HMP and HTP respectively.

##### 3.1.1.1 Fragmentation of HMP derivatives

Atmospheric pressure chemical ionization is often the method of choice for underivatized steroids, due to their low proton/electron capture and susceptibility to ion suppression in ESI mode [2], but more intense signals from ions of the derivatives were recorded with ESI, typical of species with pre-existing charge. The precursor ions of HMP derivatives were the expected molecular ions, *m/z* 394 (testosterone, EpiT, DHEA and DHA), *m/z* 392 (androstenedione) and *m/z* 396 (DHT) (Fig. 2) [21]. Two product ions of significant abundance were generated from cleavage of the *N*-*N* bond of the hydrazone (Fig. 2), specifically the 1-methylpyridinoamino moiety at *m/z* 109 [*N*-methylpyridine+NH_2_]^+^, (C_6_H_9_N_2_) for DHEA (with derivative formation on the D-Ring) and *m/z* 108 [*N*-methylpyridine+NH]^+^, (C_6_H_8_N_2_) for all other A-ring derivatives (Table 1, Supplementary Fig. S1). HMP derivatization for di-oxosteroids (androstenedione and DHA) was not as efficient as mono-oxosteroids (testosterone, DHT, EpiT and DHEA) due to formation of multiple products as opposed to just the *bis*-HMP derivative. Ultimately mono-HMP derivatives were selected and the nature of the product ions confirmed using high resolution MS (Fig. 2).

##### 3.1.1.2 Fragmentation of HTP derivatives

The precursor ions of HTP derivatives were the expected molecular ions, *m/z* 449 (testosterone, EpiT, DHEA and DHA), *m/z* 447 (androstenedione) and *m/z* 451 (DHT) (Table 2) [14] and two product ions of significant abundance were generated at *m/z* 257 (C_11_H_12_N_4_F_3_) and *m/z* 269 (C_12_H_12_N_4_F_3_) for testosterone, androstenedione and EpiT resulting from the cleavage of the steroid A and B ring (Fig. 3) [14,46]. Product ions for DHT were monitored at *m/z* 288 (C_19_H_30_ON) and *m/z* 164 (C_5_H_5_N_3_F_3_) whereas, *m/z* 286 (C_19_H_28_ON) and *m/z* 164 (C_5_H_5_N_3_F_3_) were selected for DHA and DHEA respectively. Heterolytic cleavage of hydrazine *N*-*N* bond of derivative may form ions at *m/z* 288 and *m/z* 286, and *m/z* 164 characterizes the protonated trifluoromethyl-pyrimidine moiety. Ions obtained following collision induced dissociation of derivatives of stable-isotopically labeled testosterone, androstenedione and DHT corroborated potential fragmentation (Fig. 3).

#### 3.1.2 Chromatographic resolution of androgens using HMP and HTP derivatives

##### 3.1.2.1 HMP derivatives

HMP derivatization forms two *E* and *Z* isomers which elute as double peaks for each androgen [21] creating chromatographic complexity; indeed more than ten isomers for HMP derivatives had to be separated. To overcome chromatographic complexity, initially reduction of the imines to a single component using sodium triacetoxyborohydride [47] and sodium borohydride was assessed (Supplementary Methods). This proved unsuccessful with poor yield. While this concept still has potential, chromatographic separation of isomers was pursued.

**Fig. 4:**
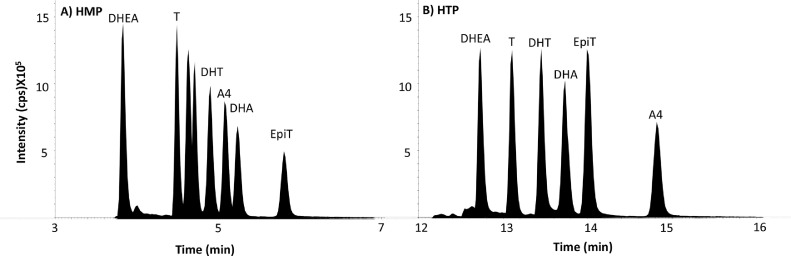
Total ion chromatograms of quantifier mass transitions of HMP and HTP derivatives; (A) HMP (DHT-HMP *m/z* 396→108; T-HMP, DHA-HMP and EpiT-HMP *m/z* 394→108; A4-HMP *m/z* 392→108; DHEA-HMP *m/z* 394→109) and (B) HTP (DHT-HTP *m/z* 451→288; T-HTP and EpiT-HTP *m/z* 449→257; A4-HTP *m/z* 447→269; DHA-HTP *m/z* 449→286; DHEA-HTP *m/z* 449→286), testosterone (T), androstenedione (A4), 5α-dihydrotestosterone (DHT), 5α-dihydroandrostanedione (DHA), epitestosterone (EpiT), dehydroepiandrosterone (DHEA), 2-hydrazino-1-methylpyridine (HMP), 2-hydrazino-4-(trifluoromethyl)-pyrimidine (HTP) and counts per second (cps).

**Fig. 5:**
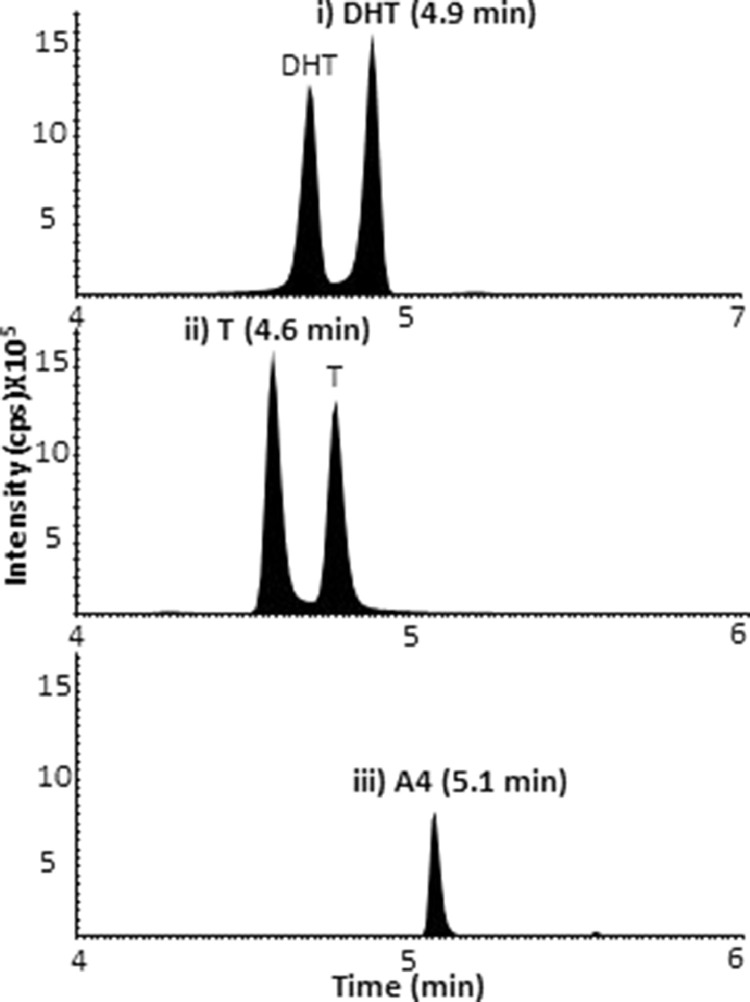
Retention times of quantifier mass transitions of HMP derivatives; (i) DHT-HMP (4.9 min), (ii) T-HMP (4.6 min) and (iii) A4-HMP (5.1 min). Testosterone (T), androstenedione (A4), 5α-dihydrotestosterone (DHT), 2-hydrazino-1-methylpyridine (HMP) and counts per second (cps).

Chromatographic analyses were developed following extensive assessment of C_18_ columns with modified stationary phases, particle sizes and dimensions. SunFire® C_18_ (2.1 mm x 150 mm, 3.5 μm), ACE® Excel PFP-C_18_ (2.1 mm x 100 mm, 3.5 μm), ACE® UltraCore 2.5 SuperC_18_ (2.1 mm x 150 mm, 2.5 μm), ACE® Excel super C_18_ (2.1 mm x 150 mm, 2 μm), Acquity UPLC® BEH C_18_ (2.1 mm x 150 mm, 1.7 μm) and Acquity UPLC® BEH C_18_ (2.1 mm x 50 mm, 1.7 μm) were evaluated aiming to achieve baseline resolution of isomers and isotopologues within reasonable analytical times. Ultimately rapid chromatographic separation was achieved between isomeric HMP derivatives of each androgen and their endogenous isomers using a UPLC® BEH C_18_ (2.1 mm x 50 mm, 1.7 μm) column operated at 50°C, with run times of 10 min (Fig. 4A). Binary organic solvents gave better resolution of testosterone and DHT derivatives than single organic solvents. The beneficial effect of adjusting acetonitrile/methanol proportion on peak separation may be due to acetonitrile influencing Π−Π and/or dipole-dipole interactions between the analyte and the column stationary phase [48,49]. Fig. 5i–iii show the peaks selected for quantitation, where notably the earlier peak for testosterone-HMP was distinct from the later peak DHT-HMP. Androstenedione-HMP isotopologues had potential to interfere with testosterone analysis but again were chromatographically resolved. At early and late time points the flow was diverted to waste and a rapid increasing organic gradient was employed latterly; both strategies minimized build-up of derivatization reagent on the column and source, required for robustness during larger batch analysis. Minimal drift in retentions times were observed throughout analytical runs (<0.05 mins throughout runs).

##### 3.1.2.2 HTP derivatives

Single peaks were observed for each HTP-analyte as reported previously [14] offering a simpler chromatographic challenge than HMP. Baseline resolution between HTP derivatives of androgens and their endogenous isomers was most readily achieved using a C_18_ UPLC column (2.1 mm x 150 mm, 1.7 μm) and gradient method (Fig. 4B) but run-times were longer than for HMP derivatives. Similar cleaning strategies were employed to the HMP method. While run time might have been reduced further, the intensities of signals of each derivative were first compared to identify the superior approach for development.

#### 3.1.3 Preliminary comparison of limits of detection and quantitation of derivatives

HMP and HTP derivatives were compared by assessing the amounts of standards yielding SNR of 3 and 10, to initially estimate LOD and LOQ respectively. Using these measures, the estimated LOD for HMP derivatives of unextracted androgen standards were 0.2, 0.4 and 0.2 pg on column (equivalent to 5, 10 and 5 pg/mL, if extracting 100 μL) and the estimated LOQs were 0.4, 0.8 and 0.4 pg (equivalent to 10, 20 and 10 pg/mL, if extracting 100 μL) on column for DHT, testosterone and androstenedione respectively. Thus, estimated LOQs were improved ~21-fold and ~ 12.5-fold for HMP-DHT and HMP-androstenedione respectively in comparison to our LC-MS/MS method for underivatized androgens [50]. However, an improvement in sensitivity was not observed for HMP-testosterone. Higher LODs were estimated for HTP (40, 21 and 52 pg on column; 1000, 500 and 1300 pg/mL, if extracting 100 μL), with estimated LOQs of 96, 54 and 83 pg on column (2400, 1285 and 2075 pg/mL, if extracting 100 μL) for DHT, testosterone and androstenedione respectively. Therefore, the HMP derivative offering higher sensitivity was selected for validation, despite the more challenging chromatographic conditions, but harnessing the benefit of shorter run times.

### 3.2 Assay validation: DHT, testosterone and androstenedione

#### 3.2.1 Recovery of androgens from matrix and assessment of matrix effects

Recovery of analytes by extraction was maximized and matrix effect minimized to ensure the cleanest possible sample to be prepared for analysis, and thus maximising column life-span and limiting source contamination. Analytes were well retained on lipophilic polymeric adsorbent Oasis® HLB [39,50]. and excellent recoveries were achieved, assessing from water initially (mean ±RSD; testosterone 97±4%, androstenedione 96±8%, DHT 96±7%, ^13^C_3_-T 97±9%, ^13^C_3_-A4 99±1% and ^13^C_3_-DHT 98±5%). Extraction from plasma was assessed using stable-isotope labeled standards as surrogates, showing similar results (mean ±RSD; ^13^C_3_-T 83±3%, ^13^C_3_-A4 86±7% and ^13^C_3_-DHT 84.4±4%; desired >80%). Ion suppression of internal standards was also within acceptable limits (mean ±RSD; ^13^C_3_-T 87±2%, ^13^C_3_-A4 89±2% and ^13^C_3_-DHT 88±6%; desired >80%). Establishing conditions which limited ion suppression reduced the potential for variability in quantitation brought about by impact of subtle variations in matrix composition, particularly for low abundance analytes such as DHT. Minimal differences (<0.05 mins) in retention time between ^13^C_3_ IS and endogenous steroid due to the presence of heavy isotope were recorded. The assessment of the whole process also demonstrated that the presence of matrix did not adversely affected derivatization efficiency.

#### 3.2.2 Selectivity of HMP derivatives

Interferents close to the retention times of the peaks of interest were not observed visually. The ratios of peak areas of quantifier to qualifier mass transitions ratios in standards (testosterone 2.1, androstenedione 1.9, DHT 2.0, ^13^C_3_-T 1.7, ^13^C_3_-A4 1.8, and ^13^C_3_-DHT 2.7), were comparable to those in plasma, showing differences less than 20% (testosterone 11%, androstenedione 13%, DHT 14%, ^13^C_3_-T 16%, ^13^C_3_ -A4 18%, and ^13^C_3_ -DHT 17%).

#### 3.2.3 Linearity of response of HMP derivatives

^13^C_3_ labeled androgens were selected as the most appropriate internal standards, since loss of deuterium may happen during derivatization, due to keto-enol tautomerism in the A-ring. Standard curves of androgen HMP derivatives from aqueous standards in the range 1-200 pg achieved acceptable linearity with a weighting of 1/x. Calibration data from in-house standard solutions were compared with those obtained using certified reference standards and found acceptable across the calibration range for androstenedione and DHT. For testosterone, the bias against certified standards was unacceptable at the lowest concentration (predicted LOQ), but acceptable within the reference range (Tables 3 and 5). We conclude certified standards should be employed moving forward, particularly in settings where androgens may be in low abundance such as post-menopausal women. Parallelism was also evaluated against a steroid-depleted serum enriched with endogenous analytes across the range ultimately measured in Table 5 and find close agreement in gradients of calibration of lines of +6.4%, -5.6 % and +8% for DHT, testosterone and androstenedione respectively.

**Table 3:** Bias of the calibrators against certified standards for testosterone (T), androstenedione (A4) and 5α-dihydrotestosterone (DHT).

Bias (%)			
Amount (pg)	T	A4	DHT
5	36	10	18
10	21	15	8
20	11	5	16
40	14	5	2
50	14	11	6
100	5	3	8
200	2	4	3

#### 3.2.4 Precision and trueness of quantitation of HMP derivatives

LODs and LOQs measured through replicate calibration lines of testosterone, androstenedione and DHT extracted from water were 0.9, 0.4 and 1.5 pg and 2.8, 1.2, and 4.6 pg on column respectively. The values of intra- and inter-assay precision and trueness for measurements were therefore assessed in replicate standards at the lowest predicted value (1 pg), intra and inter assay deemed acceptable (<20% RSD for precision and trueness at the LOQ (Table 4) and <15% above this value) for extracts from water and plasma from post-menopausal females and males. Acceptable injector repeatability of HMP derivatives was demonstrated with RSD (1 pg); testosterone (12%), androstenedione (14%) and DHT (13%).

**Table 4:** Precision and trueness of analysis of HMP derivatives of androgens. Testosterone (T), androstenedione (A4), 5α-dihydrotestosterone (DHT), Relative Standard Deviation (RSD), Relative Mean Error (RME).

Standard solutions	Low (1 pg)				Middle (50 pg)			High (200 pg)			
Analyte	T	A4	DHT		T	A4	DHT	T	A4	DHT	
Precision (%RSD) (*n=6*)											

Intra-assay	6	8	7		2	2	3	2	1	1	
Inter-assay	10	11	12		5	4	5	7	6	2	

Trueness (%RME) (*n=6*)											

Intra-assay	9	10	10		4	5	6	3	2	1	
Inter-assay	13	14	15		7	8	10	7	6	5	

Precision (%RSD) *(n=6)*											

Plasma		Male					Post-menopausal female				
Analyte		T	A4	DHT			T	A4			DHT

Intra-assay		3	3	4			4	4			5
Inter-assay		8	7	9			9	8			10

#### 3.2.5 Stability of HMP derivatives

The HMP derivatives demonstrated acceptable stability upon storage in an auto-sampler (10°C) over 24 h, with limited degradation measured for derivatized testosterone (9%, 8%), androstenedione (5%, 6%) and DHT (8%, 7%) in extracts of plasma from male and post-menopausal female subjects, respectively. Derivatives were stable upon short-term storage in the freezer (-20 and -80°C, respectively) for 7 days; reduction from original response testosterone (5%, 4%), androstenedione (8%, 7%) and DHT (9%, 8%) in extracts of samples from males and testosterone (4%, 5%), androstenedione (7%, 8%) and DHT (8%, 9%) from post-menopausal female. Moreover, derivatives were stable upon long-term storage in the freezer (-20 and -80°C, respectively) for 30 days; reduction from original response testosterone (4%, 3%), androstenedione (7%, 6%) and DHT (8%, 7%) in extracts of samples from males and testosterone (3%, 4%), androstenedione (6%, 7%) and DHT (7%, 8%) from post-menopausal females.

### 3.3 Method application

**Fig. 6:**
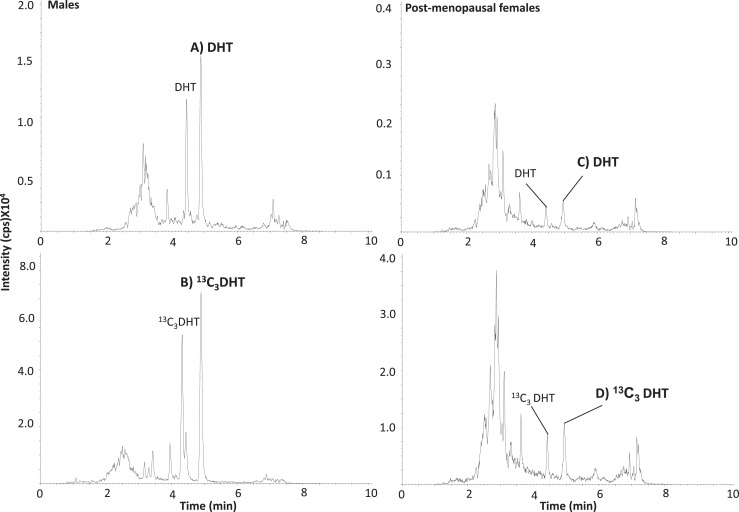
Mass chromatograms of quantifier mass transitions of DHT-HMP (*m/z* 396→108) and internal standard, ^13^C_3_-DHT-HMP (*m/z* 399→108) derivatives in extracts of plasma from Males (A and B) and post-menopausal females (C and D) respectively. 5α-dihydrotestosterone (DHT) was detected at levels of 30 pg and 14 pg/sample ((1.0 nmol/L and 241 pmo/L) in males and post-menopausal female plasma samples respectively. 2,3,4-^13^C_3_-5α-dihydrotestosterone (^13^C_3_-DHT) was added as 100pg. 2-Hydrazino-1-methylpyridine (HMP) and counts per second (cps).

The method was applied to samples from post-menopausal females and males and compared to typical reference ranges (Table 5) [1,33]. Chromatographic separation for DHT-HMP derivatives and their endogenous isobars was achieved (Figs. 5 and 6) and all concentrations were within the quantitation limits of the assay. In the dynamic research setting, frequent sampling can happen, with limitations on blood letting, emphasizing the benefits of low volume assays. In post-menopausal females, 200 µL of plasma was required to ensure reliable detection of DHT, whereas only 100 µL was required of plasma from males. Testosterone and androstenedione could be quantified in 20 µL samples in males, while 200 µL were required from post-menopausal females. Thus, overall sample volumes and LOQs compared favorably with previous literature for DHT derivatives for LC-MS/MS analysis. Volumes of serum or plasma from human and even mice in previous MS methods [3,14,27,28,35,37,51,52] were 100 - 500 µL with LOQs typically 0.2–10 pg on column. For example, 500 µL of male serum had been previously required to detect underivatized DHT in male plasma [50], whereas 300 µL of male rat plasma had been used to quantify underivatized testosterone and DHT [16]. A 2-dimensional LC system allowed online purification and separation prior to quantitation of testosterone and androstenedione in 100 µL human adult male serum [13] and for DHT in 300 µL human adult male and female serum [53]. However, on-line solid phase extraction SPE is not commonly available, although undoubtedly achieves high performance. Using smaller volumes of sample offers the advantage of a cleaner extract, less build up on the instrument, smaller SPE cartridges, therefore, less cost and time as well as opening opportunities in the animal or pediatric setting.

**Table 5:** Concentrations of androgens in plasma from males and post-menopausal females. testosterone (T), androstenedione (A4) and 5α-dihydrotestosterone (DHT).

Group	N	Age (y)	HMP derivatives (nmol/L)			reference range (nmol/L)		
			T	A4	DHT	T	A4	DHT
Males	42	21-85	4.5-21	1-5.5	0.4-1.9	8-33⁎	1.4-5.2⁎	0.4-3.0⁎
Post-menopausal females	17	58-60	0.4-1.1	0.7-2.3	0.1-1	0.4-1.1#	0.8-2.0#	<0.4⁎

⁎ Mayo clinic reference ranges accessed 29/9/2020; # [55].

## 4 Conclusions

In summary derivatization to form HMP derivatives of androgens, in conjunction with LC-MS/MS, is suitable for quantitative analysis of androgens in low abundance in biological fluids, offering advantages in sensitivity over analysis of underivatized DHT. The approach allows concomitant analysis of testosterone, androstenedione and DHT and this method may be extended to include further steroids with ketone functions e.g. 11-oxygenated androgens. Robust measurement was achieved across typical physiological ranges found in post-menopausal women and men offering an attractive alternative to immunoassays, which lack selectivity in the lower physiological range of concentrations and typically report only one androgen**.** While multi-dimensional detection in conjunction with chromatography brings anticipated benefits in selectivity [54], only a limited number of matrix samples were studied. The method should now be applied in larger populations, of both sexes, across wider age ranges and including both healthy and diseased individuals to address population wide variations in plasma composition to enable the assay to be rolled out for wider use [55]. Due to the endogenous nature of the analytes, steroid-depleted serum was used to assess parallelism on calibration, but enrichment of biological matrix from the specific subject cohorts is recommended as blood composition can vary substantially in health and disease and between species.

## CRediT authorship contribution statement

**Abdullah MM Faqehi:** Methodology, Formal analysis, Project administration, Resources, Validation, Writing - original draft. **Scott G Denham:** Methodology, Formal analysis. **Gregorio Naredo:** Methodology, Formal analysis, Writing - review & editing. **Diego F Cobice:** Methodology, Formal analysis, Writing - review & editing. **Shazia Khan:** Methodology, Formal analysis, Writing - review & editing. **Joanna P Simpson:** Methodology, Formal analysis, Writing - review & editing. **Ghazali Sabil:** Methodology, Formal analysis, Writing - review & editing. **Rita Upreti:** Investigation, Writing - review & editing. **Fraser Gibb:** Investigation, Writing - review & editing. **Natalie ZM Homer:** Methodology, Validation, Project administration, Supervision, Writing - review & editing. **Ruth Andrew:** Conceptualization, Methodology, Validation, Funding acquisition, Project administration, Supervision, Writing - review & editing.

## Declaration of Competing Interest

The authors declare that they have no known competing financial interests or personal relationships that could have appeared to influence the work reported in this paper.

## Appendix Supplementary materials

Image, application 1

Image, application 2
