# Supplementary material for: Derivatization with 2-hydrazino-1-methylpyridine enhances sensitivity of analysis of 5α-dihydrotestosterone in human plasma by liquid chromatography tandem mass spectrometry
Source: J Chromatogr A. 2021 Mar 15;1640:461933. doi: 10.1016/j.chroma.2021.461933 (PMC7938423; doi:10.1016/j.chroma.2021.461933)

**Supplementary Figure S1.** **Further Product Ion Scans of HMP derivatives and analytes and internal standards;** testosterone (T), androstenedione (A4), 5α-dihydrotestosterone (DHT), 5α-dihydroandrostanedione (DHA), epitestosterone (EpiT), dehydroepiandrosterone (DHEA), 2,3,4-^13^C_3-_testosterone (^13^C_3_-T), 2,3,4-^13^C_3_-androstenedione (^13^C_3_-A4), 2,3,4-^13^C_3_-5α-dihydrotestosterone (^13^C_3_-DHT) and 2-hydrazino-1-methylpyridine (HMP).


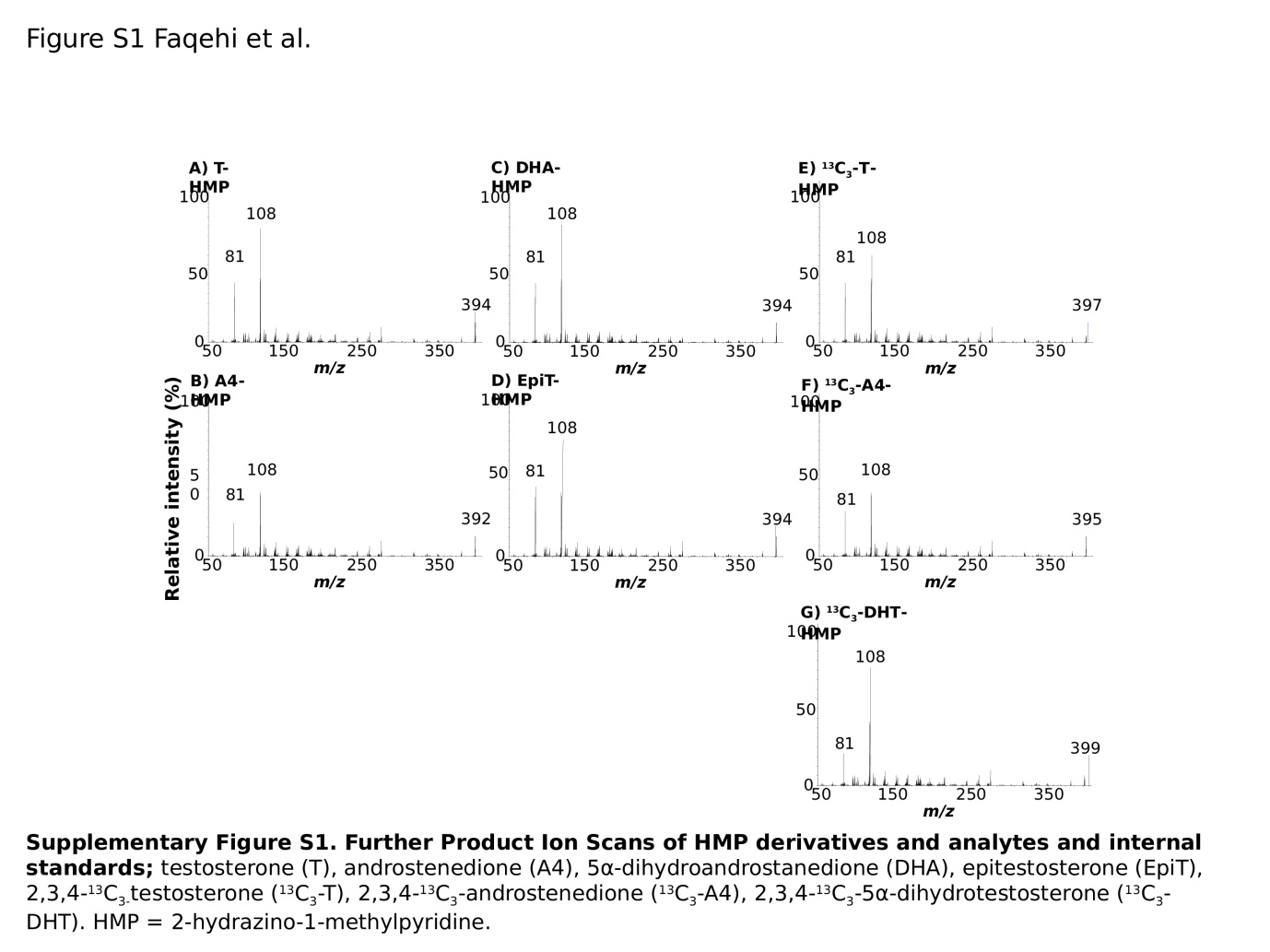

Supplement: Supplementary file 1 [file mmc1.docx]
