# Supplementary material for: Derivatization with 2-hydrazino-1-methylpyridine enhances sensitivity of analysis of 5α-dihydrotestosterone in human plasma by liquid chromatography tandem mass spectrometry
Source: J Chromatogr A. 2021 Mar 15;1640:461933. doi: 10.1016/j.chroma.2021.461933 (PMC7938423; doi:10.1016/j.chroma.2021.461933)
Supplement: Supplementary file 2 [file mmc2.docx]

**Supplementary Methods**

**Reduction of the HMP Derivatives**

Reduction of HMP imines to reduce the number of chromatographic peaks was assessed using sodium triacetoxyborohydride (NaBH(OAc)_3_) and sodium borohydride (NaBH_4_). Reducing reagents (5 µL; 4 mg/mL in methanol) were freshly prepared and added to the HMP residues (10 µg steroid equivalent). The mixture was vortexed (10 s) then incubated (60 °C, 15 min). Solvent was evaporated (OFN, 60 °C) and the residue reconstituted in mobile phase (50 µL) as above (2.6). Reducing conditions were evaluated including incubation temperature (25-80 °C), time (5-60 min), reaction volume (5-200 µL) and injection volume (1-20 µL). Potential reduced HMP derivatives were injected into the UHPLC-MS system and compared with unreduced derivatives. Molecular ions of reduced derivatives with a mass increment of 2 were monitored, predicted to elute as single peaks.
